# Differentiation of Human Scalp Adipose-Derived Mesenchymal Stem Cells into Mature Neural Cells on Electrospun Nanofibrous Scaffolds for Nerve Tissue Engineering Applications

**DOI:** 10.22074/cellj.2018.4898

**Published:** 2018-03-18

**Authors:** Mehrafarin Fesharaki, Shahnaz Razavi, Laleh Ghasemi-Mobarakeh, Mohaddeseh Behjati, Reyhaneh Yarahmadian, Mohammad Kazemi, Hejazi Hossein

**Affiliations:** 1 Department of Cell Sciences Research Center Medical Sciences, School of Medicine, Isfahan University of Medical Sciences, Isfahan, Iran; 2 Department of Anatomical Sciences, Medicine School, Isfahan University of Medical Sciences, Isfahan, Iran; 3 Department of Textile Engineering, Isfahan University of Technology, Isfahan 84156-83111, Iran; 4 Rajaie Cardiovascular Medical and Research Center, Iran University of Medical Sciences, Tehran, Iran; 5 Department of Materials Engineering, Isfahan University of Technology, Isfahan, Iran; 6 Department of Genetics and Molecular Biology, School of Medicine, Isfahan University of Medical Sciences, Isfahan, Iran; 7 Skin Diseases and Leishmaniasis Research Center, Department of Parasitology and Mycology, School of Medicine, Isfahan University of Medical Sciences, Isfahan, Iran

**Keywords:** Differentiation, Neuron, Stem Cells, Tissue Engineering

## Abstract

**Objective:**

This study aimed to isolate and culture SADS cells, investigate their neurogenic capacity and evaluate their application for nerve tissue engineering.

**Materials and Methods:**

In this experimental study, SADS cells were isolated from human adipose tissue. After 7-day treatment of SADS cells with insulin, indomethacin and isobutylmethylxanthine, neurogenic differentiation of SADS cells was investigated. During this study, Poly (ε-caprolactone) (PCL) and PCL/gelatin nanofibrous scaffolds were fabricated using electrospinning and subsequently nanofibrous scaffolds were coated with platelet-rich plasma (PRP). SADS cells were also seeded on nanofibrous scaffolds and neurogentic differentiation of these cells on nanofibers was also evaluated. Effect of PRP on proliferation and differentiation of SADS cells on scaffolds was also studied.

**Results:**

Our results showed that after 7-day treatment of SADS cells with insulin, indomethacin and isobutylmethylxanthine, SADS cells expressed markers characteristic of neural cells such as nestin and neuron specific nuclear protein (*NEUN*) (as early neuronal markers) as well as microtubule-associated protein 2 (*MAP2*) and neuronal microtubule-associated (*TAU*) (as mature neuronal markers) while mature astrocyte maker (*GFAP*) was not expressed. MTT assay and SEM results showed that incorporation of gelatin and PRP into the structure of nanofibrous scaffolds has a significant positive influence on the bioactivity of scaffolds. Our results also showed neurogentic differentiation of SADS cells on scaffolds.

**Conclusion:**

Our results demonstrated that SADS cells have potential to differentiate into early and mature progenitor neurons, *in vitro*. PCL/gelatin/PRP was found to be a promising substrate for proliferation of SADS cells and differentiation of these cells into neural cells which make these scaffolds a candidate for further *in vivo* experiments and suggest their application for nerve tissue engineering.

## Introduction

So far, the only way to replace disease or injury-induced loss of neural tissue has been through cell transplantation. Neural tissue engineering is continuously been applied as a new choice for nervous system repair and regeneration and is composed of a biomaterial-based substrate that incorporates cells and biochemical cues (1-4).

Neural progenitor cells are the important and vital components of any strategy that is employed to replace neural tissue. Long-term neural integration, regeneration and renovation require a successive supply of neural progenitor cells to be able to differentiate into neurons and glial cells (5). Mesenchymal stem cells (MSCs) are found in virtually all organs of the body and have self-renewal ability and multi- lineage capacity to differentiate into different tissue cells such as bone, cartilage, muscle, adipocytes and neural progenitors (6, 7). 

Bone marrow-derived MSCs were find first, and considered the primary source of MSCs for clinical use. Subsequently, MSCs were isolated from various other sources such as adipose tissue, serving as one of the alternatives to bone marrow as it is safer and easier to use as compared to bone marrow-derived MSCs and share many biological characteristics (7).

Recently, interest has rapidly grown in the developmentalplasticity and therapeutic potential of these cells (8-11). Previous studies showed that the human scalp tissue containsmultipotent stem cells with the capacity to differentiate alongmesodermal and ectodermal lineages (12). Adipose tissueof scalp originates from neural crest and previous studiesdemonstrated that stem cells with neural crest origin canbetter differentiate to neural linage. In this regard, Shih et al. (12) showed ectodermal neurogenic differentiation potential of stem cells available in scalp tissue.

Tissue engineering treatments, using scaffolds and livingcells exploit new advancements in understanding of thedevelopmental and cell biology that controls and directs cellfunction with the eventual aim of human tissue regenerationand repair (13). The cells which especially in current years, have been used in tissue engineering are stromal stem cells and a wide range of biomaterials has been used forfabrication of scaffolds. It is expected that scaffolds mimicthe architecture of extracellular matrix (ECM) as much aspossible and provide appropriate microenvironment for cell growth and differentiation.

Recently, nanofibrous scaffolds with the ability to mimicthe native ECM along with their high surface-to-volumeratio, interconnected pores and high porosity have attracted much interest in tissue engineering (14, 15). The usage ofelectrospinning, an operationally simple, inexpensive andversatile approach has been exponentially increased for fabrication of nanofibrous scaffolds and has been used to fabricate bio-composite nanofibers scaffolds to providemechanical support and direct the growth of different cells (1618). Prabhakarn et al. (18) showed neuronal differentiation ofhuman bone marrow derived MSCs on electrospun poly(llactic acid)-co-poly-(ε-caprolactone)/Collagen (PLCL/Coll) nanofibrous scaffolds.

Poly (ε-caprolactone) (PCL) is a semi crystalline linearhydrophobic polymer. Although, the electrospun PCL nanofibrous scaffolds mimic the dimension of ECM in living tissues, its hydrophobic nature reduces the ability ofcell adhesion, migration, proliferation and differentiation, necessary for tissue differentiation. Our previous studyshowed that incorporation of gelatin into PCL nanofibrousscaffolds increases the hydrophilicity of scaffolds leading tohigher rates of cell attachment and proliferation on resultant nanofibrous scaffolds (19).

Moreover, platelet-rich plasma (PRP) is described as plasma with the platelet population of >1.0×10^6^ cells/µl thatcontains various growth factors such as trans forming growthfactor (TGF), platelet-derived growth factor (PDGF), plateletsderived-epidermal growth factor (PDEGF), platelet-derivedangiogenesis factor (PDAF), insulin growth factor -1 (IGF1) and vascular endothelial growth factor (VEGF). PRP canbe considered an autologous healing biomaterial and appliedto accelerate cell proliferation and matrix synthesis in tissueengineering. Moreover, its availability, cost-effectiveness, wide range of applications, and autologous feature make it suitable for several clinical applications (20-24). 

The purpose of the present study was to isolate and culture scalp adipose-derived MSCs (SADS cells), evaluate their neurogenic capacity and also discuss the possibility of application of SADS cells in nerve tissue engineering, as apreliminary study. During this study, nanofibrous scaffoldswere coated with PRP to examine the effect of PRP on cell proliferation and morphology. 

## Materials and Methods

### Cell isolation and culture

In this experimental study, scalp adipose tissue was obtainedfrom healthy volunteers under local anesthesia. Biopsies of 5×5 mm^2^ were obtained from healthy scalp, and transferred tocell culture laboratory in phosphate-buffered saline solution (PBS).

The fragments were washed extensively by sterile PBS atleast three times. Subcutaneous fat was manually removedwith eye scissors. The remaining tissue, was again washedwith PBS, cut into 1×1 m pieces and then cultivated intissue culture medium including DMEM/F12 and modifiedEagle’s medium (Gibco BRL, Paisley, UK) containing 12% fetal bovine serum (FBS, Gibco, UK), 1% streptomycin/ penicillin solution (CM Media, Sigma-Aldrich, USA), andincubated in a humidified incubator at 37°C with 5% CO_2_ and defined as passage 0 (P0).

Culture media was replaced every 3 days until 80% confluency was obtained. Then, cells were split using 0.05% trypsin/0.02% EDTA and sub-cultured for more passages. This process was repeated until passage 3 and cells wereused in the present study (all chemicals were obtained fromSigma, St. Louis, MO, USA, unless stated otherwise). Allexperimental procedures were approved by the Scientific andEthics Committee of Isfahan University of Medical Science, Isfahan, Iran.

### Flow cytometry

After passage three of culture, the expression of surface markers was evaluated using Monoclonal antibodies including CD44, CD90, CD105 and CD45 antibodies (BD/ Pharmingen, San Diego, CA). 

The adherent cells were detached, and re-suspended inPBS. Aliquots containing 5×10^5^ cells were incubated with primary antibodies for 15 minute at 4-8°C. Finally, the cellswere analyzed using a FACS Calibur cytometer (Becton Dickinson). For each sample, 1×10^4^ events were acquired and studied by the CELLQUEST Pro software. All events wereobtained under similar conditions and cellular debris were removed from analysis. Expression of cell surface marker was analyzed by isotype control on a histogram plot.

### Neurogenic differentiation 

SADS cells were detached using trypsin-EDTA and cultured in DMEM/F12 modified Eagle’s medium supplemented with 10% FBS, 1% penicillin/streptomycin/ antimycotic, 5 µg/mL insulin, 200 µM indomethacin and 0.5 mM isobutylmethylxanthine (Sigma-Aldrich, St. Louis). This media was labeled as NM hereafter. The media was replaced every 3 days with fresh media. Ashjian et al. (25) also used isobutylmethylxanthine, indomethacin, and insulin for differentiation of human processed lipoaspirate into early neural progenitors.

### RNA isolation and quantitative real-time polymerase chain reaction 

Total RNAwas isolated by RNeasy mini kit (Qiagen, USA), and treated with RNase free DNase set (Qiagen, USA) toeliminate the genomic DNA according to the manufacturer’sinstructions. The RNA was reverse transcribed usingRevertAid First Strand cDNA Synthesis Kit (Fermentase, USA) with oligo dT primers. The real-time polymerase chainreaction (RT-PCR) was carried out using Maxima SYBRGreen RoxqPCR master mix kit (Fermentase, USA) andStepOne Plus™ quantitative Real time PCR detection System(Applied Biosystems, USA). PCR reactions were performedat a total volume of 20 µl. The PCR amplification conditionsconsisted of 10 minutes at 95°C followed by 40 cycles ofdenaturation step at 95°C for 15 seconds and annealing andextension for 1 minute at 60°C. The relative quantification(RQ) was calculated as the ratio of the mean value of the target gene to the mean value of the reference gene (GAPDH) in each sample. The relative amount of PCR products generated from each primer set, was determined on the basis of the cycle threshold (Ct) value. The RQ was calculated by 2^-ΔΔCT^. These experiments were carried out in triplicate and independently repeated at least three times. Same method was applied for investigation of cells differentiation on scaffolds.

### Preparation of platelet-rich plasma

In this study, PRP was prepared according to method describe by Sell et al. (26). Briefly, 40 mL whole blood was obtained from Iran blood transfusion organization and added to tubes containing acid citrate- dextrose as an anti-coagulant (0.163 mL per 1 mL of blood) immediately after being drawn; blood was centrifuged at 1500 rpm for 10 minutes to separate the plasma containing the platelets from the red blood cells. The collected supernatant was centrifuged again at 3000 rpm for 10 minutes, and precipitated platelets were collected. The platelets were re-suspended in a proper volume of plasma to achieve a platelet concentration 8-10 times above the physiologic amounts.

### Preparation of nanofibrous scaffolds 

The polymer solution of PCL and PCL/gelatin (70:30) at concentrations of 11% (w/v) wt% and 6% (w/v) were prepared by dissolving PCL and PCL/gelatin in dimethyl formamide/methylene chloride (80:20 v/v) and hexafloro-2propanol, respectively and then stirred for 24 hours at room temperature. The solution was electrospun from a 5 mL syringe with a needle diameter of 0.4 mm at a mass flow rate of 1 mL/hour. A high voltage was applied to tip of the needle attached to the syringe when a fluid jet was ejected. PCL and PCL/gelatin nanofibrous scaffolds were also coated with PRP by soaking samples in PRP, overnight.

Before cell seeding, scaffolds were exposed to UV radiation for 2 hours, washed 3 times with PBS for 20 minutes each and incubated with culture media for 12 hours. PCL, PCL/gelatin, PCL/PRP and PCL/gelatin/PRP nanofibrous scaffolds were placed in a 24-well plate and SADS cells were further seeded on scaffolds at a density of 1×10^4^ cells/well with NM at 37°C, with 5% CO_2_ and 95% humidity.

### In vitro cell culture study

The morphology of SADS cells differentiated to nervecells on PCL, PCL/PRP, PCL/gelatin, PCL/gelatin/PRP wasobserved by SEM. After 7 days of cells seeding, sampleswere fixed using 3% glutaraldehyde (Sigma-Aldrich, St. Louis) for 2 hours. Specimens were rinsed with water anddehydrated using graded concentrations (50, 70, 90, and 100v/v) of ethanol. Subsequently, the samples were treated withhexamethyldisilazane (HMDS) (Fluka) and air-dried under afume hood. Finally, the samples were coated with gold for the observation of cell morphology. The cell proliferation on different substrates was determined using the colorimetric MTT assay. After 7 days of cells seeding in 24-well plate, cells were washed with PBS and then media were replacedwith a basal medium containing 0.005% MTT solution. After4-hour incubation at 37°C with 5% CO_2_, the medium was discarded and the precipitated formazan was dissolved indimethyl sulfoxide (DMSO). The plate was incubated for 30minutes and aliquots were pipetted in to a 96-well plate. Theabsorbance of each well was detected by a Micro plate reader (Hyperion MPR 4, Germany) at the wavelength of 540 nm. 

RNA isolation and quantitative real-time RT-PCR were also carried out for seeded cells on different scaffolds according to the aforementioned method (section 2.4). For RT-PCR, cells were seeded on scaffolds at a density of 2×10^5^ cells/well as more cells were needed for RT-PCR. 

### Statistical analysis

All data are presented as mean ± SD. Statistical analysis was carried out using single-factor analysis of variance (ANOVA). A P<0.05 was considered statistically significant.

## Results

### Isolation, characterization and differentiation of SADS cells 

In this study, human scalp adipose stem cells (SADS cells) were isolated from human scalp adipose tissue. SADS cells similar to processed lipoaspirate (PLA) cells, were expanded easily *in vitro* and exhibited a fibroblast-like morphology.

**Fig.1:**
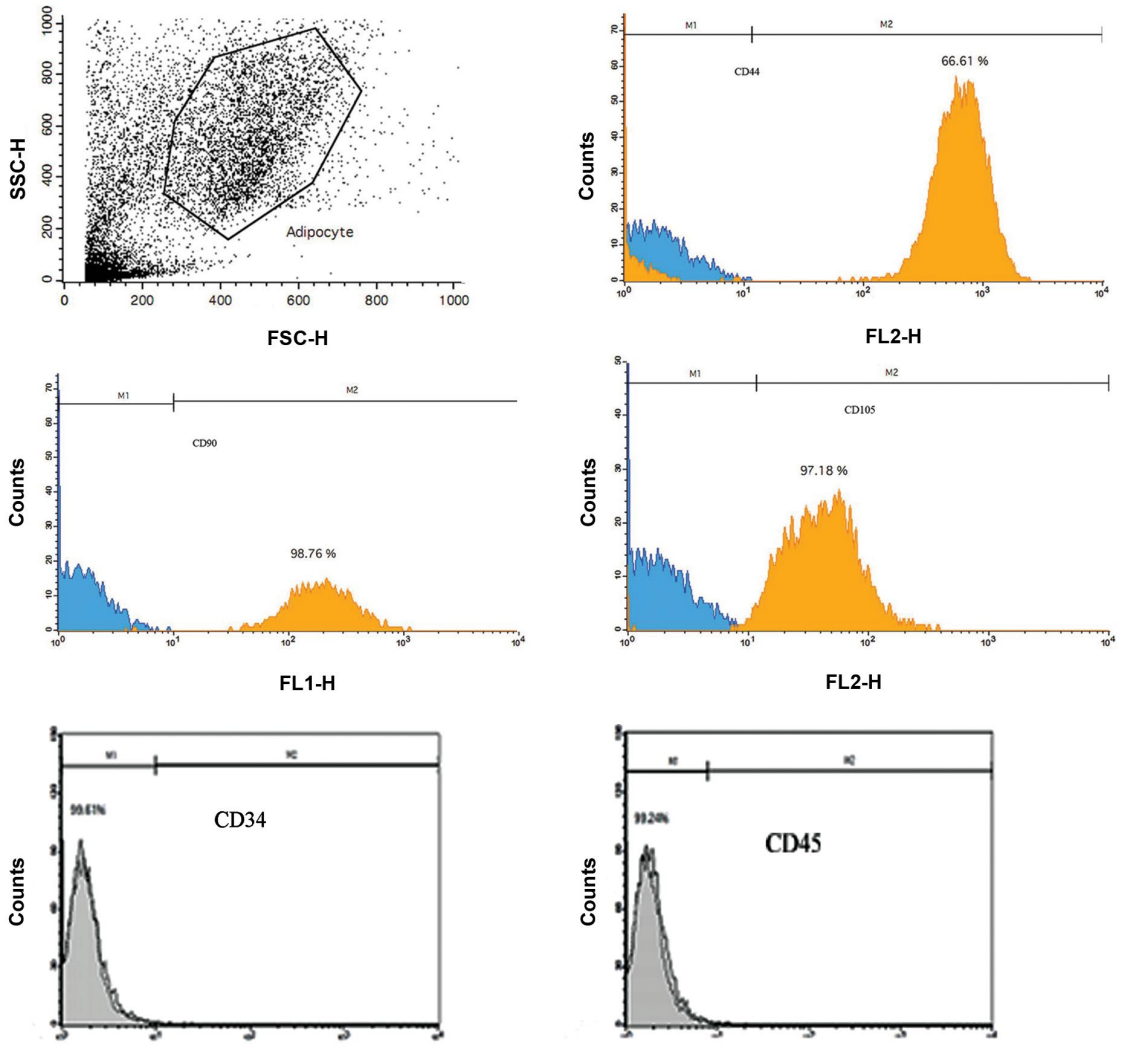
Flow cytometric analysis of SADS cells shows that human SADS cells express CD44, CD90 and CD105 but not CD34 and CD45.

In order to characterize the SADS cells, cell surface marker expression of isolated SADS cells at the third passage was analyzed. Flow cytometric analysis showed that human SADS cells do not express CD34 and CD45 but express CD90 (98.76%), CD44 (66.61%) and CD105 (97.18%) revealing adipose tissue nature of these cells (Fig .1). 

**Fig.2:**
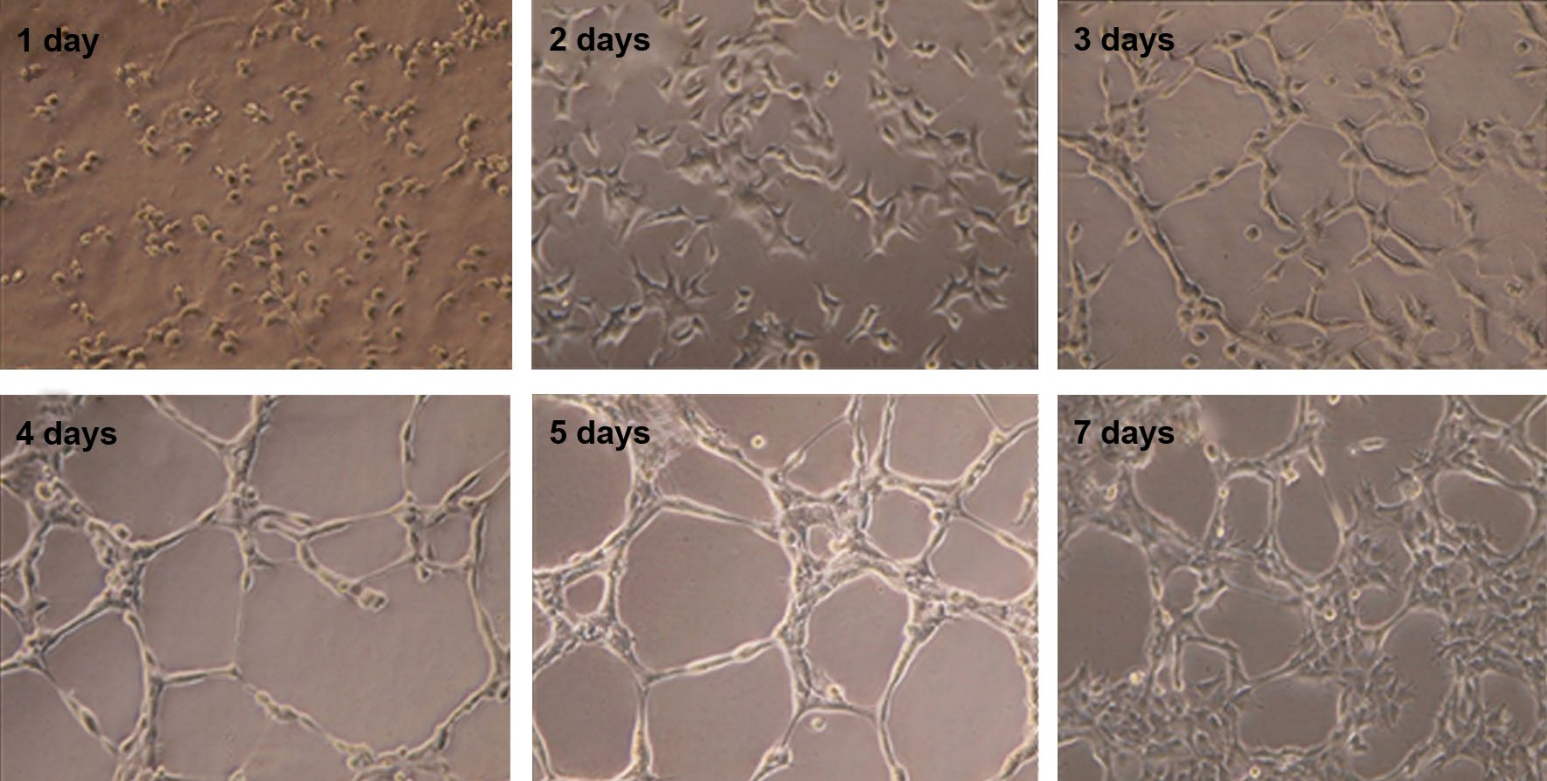
Morphology of cells cultured in NM after 1, 2, 3, 4, 5, 7 days of cell seeding (×40).

Human SADS cells were induced to differentiate in culture by incubation with NM. As early as day 2 (from day 2 to day 7) of neural induction, morphologic changes were noted. Specifically, the morphology of SADS cells changed from flat, elongated and spindle-shaped cells to rounded cells with several branching extensions and retractile characteristics (Fig .2). 

**Fig.3:**
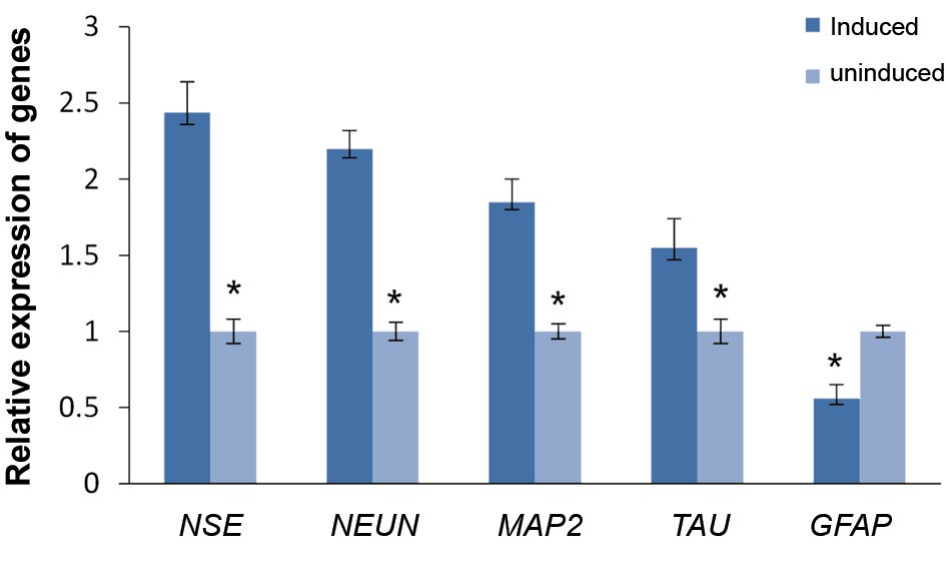
Real-time polymerase chain reaction (RT-PCR) analysis of *NES, NEUN, MAP2, TAU* and *GFAP* expression in undifferentiated and neurally induced SADS cells. *; Significance level set at P<0.05.

After 10-day treatment of SADS cells with NM, cells expressed markers characteristic of neural cells such as Nestin (*NES*) and neuron specific nuclear protein (*NEUN*) (as early neuronal markers), as well as microtubuleassociated protein 2 (*MAP2*) and neuronal microtubuleassociated (*TAU*) (as mature neuronal markers) but did not express matured astrocyte maker (*GFAP*) (Fig .3). 

### Morphology and proliferation of SADS cells on nanofibrous scaffolds 

**Fig.4:**
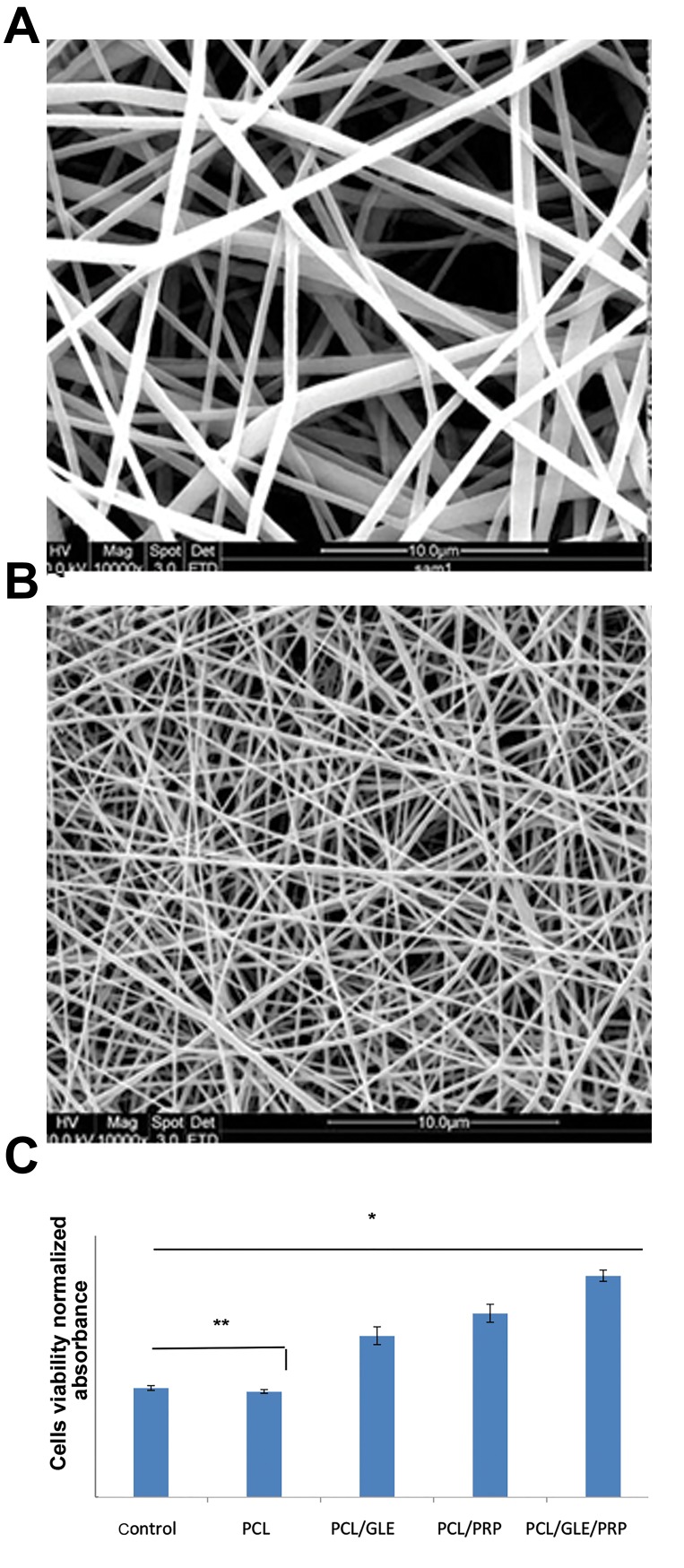
Morphology of PCL and PCL/gelatin nanofibers. Morphology of **A.** PCL and **B.** PCL/gelatin nanofibrous scaffolds, and C. MTT results of SADS cells seeded on PCL, PCL/gelatin, PCL/PRP and PCL/gelatin/PRP after 7 days of cell seeding. *; Significance set at P<0.05, **; Not significant difference (P>0.05), PCL; Poly (ε-caprolactone), and PRP; Platelet-rich plasma.

SEM micrograph of PCL and PCL/gelatin nanofibersshowed uniform and bead-free nanofibers (Fig .4). Fiber diameter was found to be 431 ± 118 nm and 189 ± 56 nm for PCL and PCL/gelatin nanofibers, respectively. PCL andPCL/gelatin nanofibers were fabricated and characterized inour previous study. More details and information regardingcharacterization of PCL and PCL/gelatin nanofibers (fiberdiameter distribution, porosity, mechanical properties, andbiodegradability) were reported in our previous study (19).

MTT assay was carried out to evaluate the proliferation of SADS cells on PCL, PCL/gelatin, PCL/ PRP and PCL/ gelatin/PRP nanofibrous scaffolds after 7 days of cell seeding. Incorporation of gelatin into the structure of PCL nanofibrous scaffolds significantly enhanced cell proliferation compared to PCL nanofibrous scaffolds without gelatin (P<0.05, Fig .4).

Coating of scaffolds with PRP was also found to increase cell proliferation whereas the proliferation of cells on PCL/ PRP and PCL/gelatin/PRP scaffolds was found to be higher in comparison to PCL and PCL/gelatin alone scaffolds (P<0.05).

**Fig.5:**
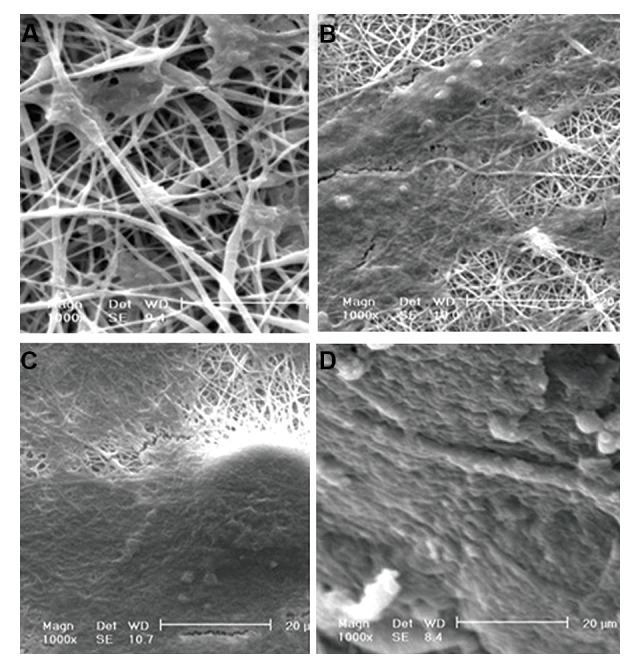
Morphology of differentiated cells on **A.** PCL, **B.** PCL/gel, **C.** PCL/PRP, and **D.** PCL/gelatin/PRP after 7 days of cell seeding on scaffold with NM (×1000). PCL; Poly (ε-caprolactone) and PRP; Platelet-rich plasma.

Morphology of cells on different scaffolds after 7 days of cell seeding revealing good integration of cells and scaffolds (Fig .5). SEM results are also consistent with MTT results and indicate higher levels of cell spreading and proliferation on PCL/gelatin nanofibrous scaffolds compared to PCL nanofibrous scaffolds. Moreover more cell spreading and proliferation was observed on scaffolds coated with PRP compared to those without PRP.

**Fig.6:**
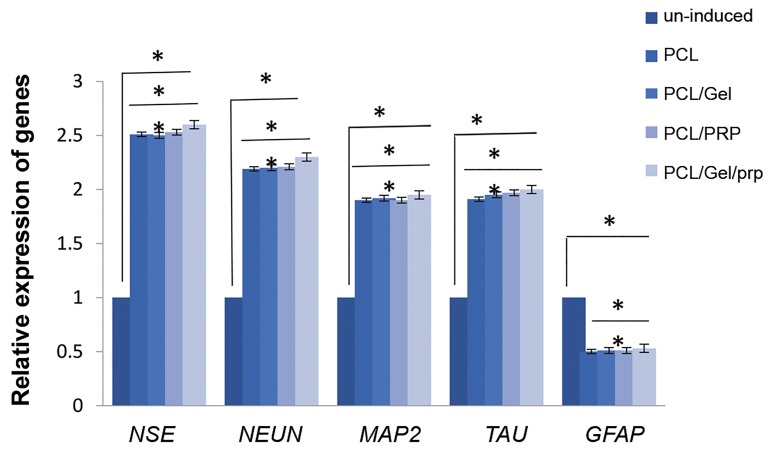
Real-time polymerase chain reaction (RT-PCR) analysis of *NES, NEUN, MAP2, TAU* and *GFAP* expression in undifferentiated and neurally induced SADS cells seeded on PCL, PCL/PRP, PCL/gelatin, PCL/gelatin/PRP. *; Significance level set at P<0.05, PCL; Poly (ε-caprolactone), and PRP; Platelet-rich plasma.

Expression of *NES, NEUN, MAP2, TAU* and *GFAP* on different scaffolds revealed differentiation of SADS cells to neural cells on nanofibrous scaffolds (Fig .6). However, no significant difference was observed in the expressionof *NES, NEUN, MAP2, TAU* and *GFAP* among differentscaffolds (P>0.05) indicating that substrate does not have anysignificant effect on differentiation of cells.

## Discussion

In this study, SADS cells were isolated from human adipose tissue of scalp; after mincing biopsies, the specimens were maintained in DMEM/F12 media supplemented with 12% FBS. We also used the media containing 10% FBS and did not observe any alteration in the morphology of cells (data not shown), while a significant increase in proliferation rate and neurogenic differentiation capacity were detected following utilization of 12% FBS. 

Flow cytometric results showed that isolated SADS cells, after the third passage were positive for CD44 (66.61%), CD90 (98.75%) and CD105 (97%) but did not express CD 34 and CD45. Other researchers have shown that bone marrow-derived stromal stem cells and adipose-derived stem cells (ADSCs) have comparable phenotype (27) and it was also reported by Zuk et al. (28) and Gronthos et al. (29) that ADSCs and MSCs are phenotypically similar as both cell populations possess CD44, CD90 and CD 105 surface markers of adipose tissue.

Several studies have established the neurogenic differentiation potential of ADSCs and MSCs. In other words, lots of studies have shown that adipose stem cells can differentiate into neural cells in the cell culture media (25, 30-32). Despite numerous studies done on ADSCs, no study inspected the probable neurogenic potential of human SADS cells, to date. Shin et al. (12) showed that human SADS cells differentiate into neuronal precursors and they suggested that these cells can be used as an alternative for neural repairing. To the best of our knowledge, it is the first research which focused on human SADS cells and demonstrated that they can differentiate into neural cells, *in vitro*. SADS cells are easily obtained, simply cultured and effortlessly expanded, *in vitro*. These cells also harvested in a safe manner with minimal risk for donors.

Ashjian et al. (25) employed induction protocol by using indomethacin, isobutylmethylxanthine, and insulin for differentiation of human PLA cells to early neural progenitors. In our study, we used the same media. They observed that cells cultured in neural induction media had an increased expression of *NSE* and *NEUN* as early markers of neurons but did not express mature astrocyte marker *(GFAP), MAP2* or *TAU* as mature neuronal markers. But, in this research, we observed that cells cultured in neural induction media had an increased expression of *NES* and *NEUN* as early markers of neurons and *MAP2* and *TAU* as mature neuronal markers. However, no expression of the mature astrocyte marker, *GFAP* was observed during this study. Our data suggest that human SADS cells may have the potential to differentiate into early and mature progenitor neural cells, *in vitro*.

PCL is a biodegradable polymer that has been used for tissue engineering applications due to its excellent mechanical properties, availability, solubility in a wide range of solvents and ability to blend with different polymers. However, due to its hydrophobic nature and lack of functional groups in its structure, the rate of cell attachment to the surface of PCL scaffolds is low (19, 32). Gelatin is a natural biopolymer derived from collagen and formed by breaking the triple-helix structure of collagen into a single-strand molecule. Moreover, the researchers found a biochemical interaction between cells and gelatin exposed to the surface of the nanofibers (17, 32). In our previous study, we investigated the effect of addition of gelatin to PCL on properties of final scaffolds and our results showed that PCL/gelatin at a weight ratio of 70:30, is suitable substrate for nerve tissue engineering application in terms of mechanical properties, biodegradation rate and cell attachment (19). 

We also applied PCL/gelatin 70:30 nanofibers as nerve guide in an *in vivo* model and our findings showed that PCL/gelatin 70:30 can serve as an appropriate substrate for peripheral nerve regeneration (33). Based on the attractive properties of PCL for biomedical applications, in this study, we modified the surface of PCL nanofibers by coating them with PRP and compared cell attachment and proliferation between PCL and PCL/PRP nanofibers. Moreover, we coated PCL/gelatin 70:30 nanofiber with PRP to examine the effect of PRP on cell behavior.

Our results showed higher proliferation of SADS cells on scaffolds containing gelatin which is consistent with previous studies. To date, using of PRP in clinical applications has attracted more attention tissue repair and regeneration with very minimal threat to the patient. The collection of whole blood, the concentration and isolation of platelets to make PRP and its application in different forms such as liquid and in lyophilized forms has been demonstrated to be effective for improving cellular activity (34-40). 

Also, PRP contains specific growth factors such as TGF-ß, PDGF, FGF, VEGF and IGF. In this study, higher proliferation rates were observed for SADS cells seeded on the scaffolds coated with PRP which is likely due to the presence of the above-mentioned growth factors in the structure of PRP. RT-PCR analysis also showed differentiation of SADS cells to neural cells on all scaffolds.

Overall, our results showed differentiation of SADS cells to early and mature progenitor neural cells on nanofibrous scaffolds. PCL/gelatin/PRP nanofibrous scaffolds can serve as a good substrate for proliferation and differentiation of SADS cells to nerve cells and act as a good candidate for further *in vivo* experiments and nerve tissue engineering applications. 

## Conclusion

We have revealed that stem cells derived from scalp adipose tissue could be isolated rapidly and simply. These stem cells were similar to other adipose-derived stem cells. Our results provide significant information regarding the optimum isolation of MSCs from adipose tissue for increasing clinical applications. Our data suggest that human SADS cells may have the potential to differentiate into early and mature progenitor of neurons, *in vitro*. Coating of nanofibrous scaffolds with PRP influenced the morphology and proliferation of SADS cells seeded on the nanofibrous scaffolds. PCL/ gelatin nanofibrous scaffolds coated with PRP were found to be the best substrate for SADS cells in terms of cell proliferation and morphology which make these scaffolds marked candidates for further *in vivo* experiments and nerve tissue engineering applications. 
